# The usefulness of anti-HCV signal to cut-off ratio in predicting hepatitis C viremia and the effect of genotype differences on signal to cut-off ratio

**DOI:** 10.1590/1806-9282.20240370

**Published:** 2024-09-02

**Authors:** Burak Sarıkaya, İrem Demiralp Yatar, Soner Yılmaz, Yasin Tiryaki, Vahibe Aydın Sarıkaya, Rıza Aytaç Çetinkaya, Duygu Kırkık

**Affiliations:** 1 University of Health Sciences, Sultan 2. Abdulhamid Han Training and Research Hospital, Department of Infectious Diseases and Clinical Microbiology – İstanbul, Turkey.; 2 Gülhane Training and Research Hospital, Regional Blood Center – Ankara, Turkey.; 3 University of Health Sciences, Sultan 2. Abdulhamid Han Training and Research Hospital, Department of Medical Microbiology – İstanbul, Turkey.; 4 Haydarpaşa Numune Training and Research Hospital, Department of Infectious Diseases and Clinical Microbiology – İstanbul, Turkey.; 5 Reyap Health Group, Department of Infectious Diseases and Clinical Microbiology – İstanbul, Turkey.; 6 University of Health Sciences, Hamidiye Medicine Faculty, Department of Medical Biology – İstanbul, Turkey.

**Keywords:** Hepatitis C virus, Genotype, Hemodialysis, Ribonucleic acid

## Abstract

**OBJECTIVE:**

In the hepatitis C virus (HCV) diagnostic algorithm, an anti-HCV screening test is recommended first. In countries with low HCV prevalence, anti-HCV testing can often give false-positive results. This may lead to unnecessary retesting, increased costs, and psychological stress for patients.

**METHODS:**

In this study, the most appropriate S/Co (signal-cutoff) value to predict HCV viremia in anti-HCV test(+) individuals was determined, and the effect of genotype differences was evaluated. Of the 96,515 anti-HCV tests performed between 2020 and 2023, 934 were reactive. A total of 332 retests and 65 patients without HCV-ribonucleic acid (RNA) analysis were excluded. Demographic data were calculated for 537 patients, and 130 patients were included in the study.

**RESULTS:**

The average age of 537 patients was 55±18 years, and 57.1% were women. The anti-HCV positivity rate was 0.62% (602/96,515), and the actual anti-HCV positivity rate was 0.13% (130/96,515). Anti-HCV levels were higher in HCV-RNA(+) patients than in HCV-RNA-negative individuals (p<0.0001) (Table 1). Receiver operating characteristic curve analysis identified the optimal S/Co value to be 10.86 to identify true positive cases. Sensitivity was 96.1%, specificity was 61.2%, positive predictive value (PPV) was 44.2%, and negative predictive value (NPV) was 98% (Figure 2). A total of 107 (82.3%) of the patients were identified as GT1, and the most common subtype was GT1b (n=100).

**CONCLUSION:**

If anti-HCV S/Co is ≥10.86, direct HCV RNA testing may be recommended; However, the possibility of false positivity should be considered in patients with a S/Co value below 10.86.

## INTRODUCTION

Hepatitis C virus (HCV) is a virus that can lead to acute and chronic hepatitis^1^. In the HCV diagnosis algorithm for HCV, the process starts with an anti-HCV screening test, and if the result is reactive, an HCV-ribonucleic acid (RNA) test is recommended for a definitive diagnosis^2,3^. As a reactivity threshold in anti-HCV tests, a value of S/Co≥1 is considered positive based on the manufacturer's recommendation^4^. In countries with low HCV prevalence, anti-HCV tests often give false positive results and slightly exceed the cutoff value^5^. Obtaining a false-positive result in anti-HCV testing may lead to unnecessary test repetitions in laboratories, increased costs due to the need for confirmatory testing, and psychological stress for patients^4^.

In this study, the primary objectives were to determine the most appropriate S/Co value for predicting HCV viremia in individuals with positive anti-HCV results and to evaluate the impact of HCV genotype differences on this prediction.

## METHODS

This study was conducted with the approval of the Health Sciences University Hamidiye Clinical Research Ethics Committee (15.02.2023/14772).

This is a retrospective descriptive study comparing the results of anti-HCV, HCV-RNA, and HCV genotype tests requested at Sultan 2. Abdulhamid Han Training and Research Hospital between 2020 and 2023. The study included patients aged ≥18 years with anti-HCV(+) results and simultaneous testing of HCV-RNA. Patients under the age of 18 years with an anti-HCV(−) result and those without simultaneous testing for HCV-RNA and genotyping were excluded.

**Figure 1:**
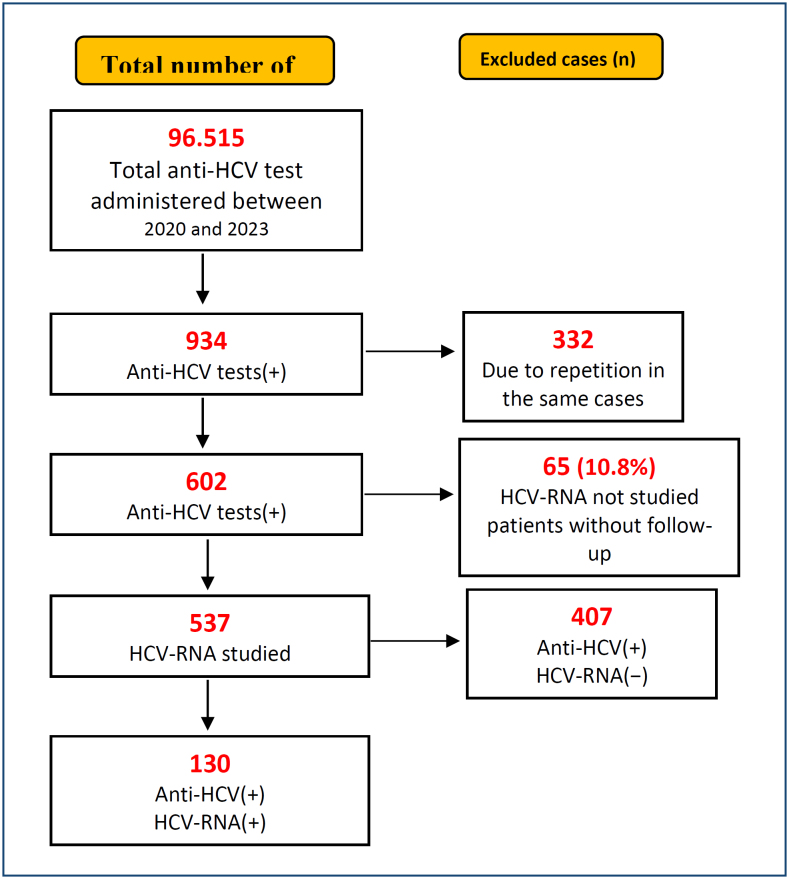
A schematic flowchart describing the inclusion and exclusion design.

A total of 96,515 anti-HCV tests were performed during the 3-year study period. As a result of exclusions, anti-HCV and HCV-RNA tests were performed simultaneously in 537 patients. Demographic data were calculated for these 537 patients. Among these patients, 130 were diagnosed with chronic HCV infection based on the detection of HCV-RNA positivity, and genotyping was performed for all of them (Figure 1).

Anti-HCV tests were performed using the "Electrochemiluminescence immunoassay" (ECLIA) method using the fourth-generation "Elecsys Anti-HCV II" kit (Roche Diagnostics, Germany), following the manufacturer's recommendations.

For the detection of HCV-RNA, viral nucleic acid isolation was conducted using the "QIAsymphony DSP virus/pathogen midi kit" (Qiagen, Germany) on the "QIAsymphony SP/AS" system, while the polymerase chain reaction (PCR) was performed using the "Artus HCV QS-RGQ" kit (Qiagen, Germany) on the "Rotor-Gene Q" system, following the manufacturer's guidelines.

In the determination of HCV genotyping, the "Geno Sen's HCV RG Genotype 1/2/3/4" qualitative real-time PCR kit (Corbett Research, Australia) and the "GEN-C 2.0 reverse hybridization strip assay" kit (NLM Diagnostics, Italy) were utilized.

### Statistical analysis

Statistical analysis was conducted using IBM SPSS version 22. The normal distribution of the data was assessed using the Shapiro–Wilks test. For difference analysis, the chi-square test was used for categorical data, the Student's t-test and ANOVA were employed for continuous data that met parametric assumptions, and Mann-Whitney U and Kruskal–Wallis tests were used for those not meeting parametric assumptions. Diagnostic accuracy was evaluated through receiver operating characteristic (ROC) curve analysis, and the cutoff values were determined using the Youden's Index. Statistical significance was investigated at a confidence level of 95% (p<0.05).

## RESULTS

The average age of the 537 patients included in the study was 55 years, with 301 (57.1%) being female (Table 1).

**Table 1:** Baseline characteristics of patients according to HCV RNA.

	All patients (n=537)	HCV-RNA(+) (n=130)	HCV-RNA(−) (n=407)	p-value
Female, n (%)	301 (57.1%)	67 (51.5%)	234 (57.5%)	0.234
Age, SD	55±18	58±17	54±19	0.023
Anti-HCV S/Co ratio	32±42	47±37	28±42	<0.0001

n: number of patients; SD: standard deviation; HCV: hepatitis C virüs; S/Co ratio: signal to cutoff ratio.

The anti-HCV positivity rate was 0.62% (602/96,515), and the actual anti-HCV positivity rate was 0.13% (130/96,515) (Figure 1).

The average age of patients in the HCV-RNA(+) group was found to be higher than that of the HCV-RNA(−) group, and this difference was statistically significant (p=0.023). When examining gender distribution, no statistically significant difference was found between the two groups of patients (p=0.234) (Table 1).

The anti-HCV levels of patients with HCV-RNA(+) results were statistically significantly higher than those with HCV-RNA(−) results (p<0.0001) (Table 1). Quantitative analysis of HCV-RNA was conducted, and a significant correlation was found between the anti-HCV S/Co ratio and HCV-RNA levels (Spearman correlation coefficient: 0.218; p=0.013).

**Figure 2:**
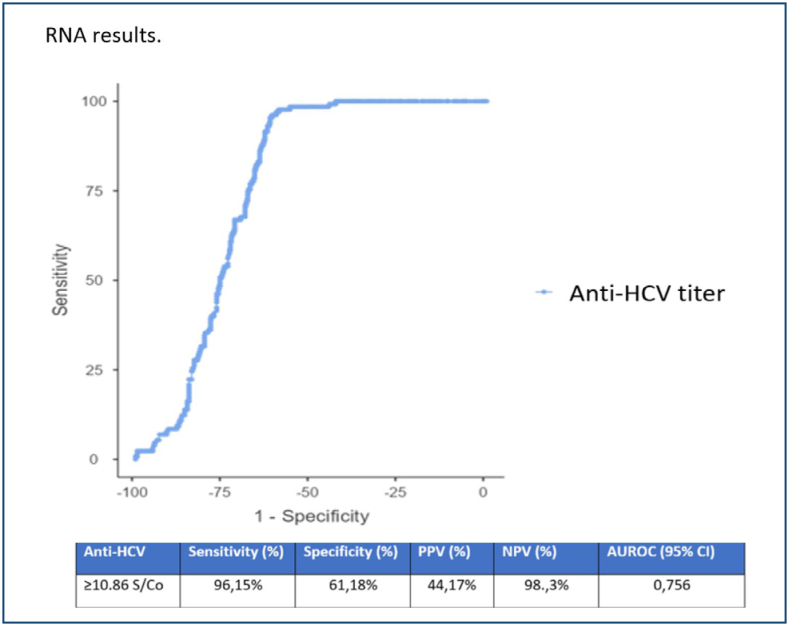
Receiver operating characteristic analysis of anti-hepatitis C virus S/Co values according to HCV RNA results.

In the ROC curve analysis, HCV-RNA was considered the gold standard to determine the best threshold value, and the most suitable S/Co value was found to be 10.86. The sensitivity was 96.1%, specificity was 61.2%, and positive predictive value (PPV): 44.2% and negative predictive value (NPV): 98% were calculated (Figure 2).

HCV genotype analysis was conducted for the entire cohort of 130 patients. Genotype-1 was identified in 107 patients (82.3%), with the most common subtype being 1b (n=100). Genotypes 5/6/7 were not detected. There were 16 patients (12.3%) with Genotype-3, one patient with Genotype-2, and four patients (3.07%) with Genotype-4. There was no statistically significant difference in the mean values of anti-HCV and HCV-RNA between those with Genotype-1 and other genotypes (p=0.759 and p=0.333). Patients with Genotype-1 were found to be older, and this difference was statistically significant (p<0.001).

## DISCUSSION

HCV infection is prevalent in all regions of the world, with the highest disease burden found in the Eastern Mediterranean and European regions^1^. While Pakistan (5.8%), Uzbekistan (4.4%), and Thailand (1.7%) have significantly higher prevalence rates, Austria (0.4%), Sweden (0.7%), Canada (0.8%), and Iran (0.4%) report lower rates^6^. Turkey is considered one of the countries with low HCV prevalence worldwide. Various studies conducted in Turkey have reported anti-HCV positivity rates ranging from 0.5 to 1.85%^5,7-9^. In our study, the anti-HCV positivity rate was found to be 0.62% (602/96,515), which is consistent with other studies.

The CDC recommends universal HCV screening, advising HCV screening at least once in a lifetime for all adults aged 18 years and older, except in settings where the prevalence of HCV infection is less than 0.1%, and for all pregnant individuals during each pregnancy^10^. Besides this recommendation, in our country, HCV screening is conducted before blood donation, before surgery, before marriage, during employment entry, and during periodic check-ups. The anti-HCV test is used for screening HCV infection, and if anti-HCV positivity is detected, a confirmatory test, the HCV-RNA test, is performed for a definitive diagnosis^8^. In countries with low HCV prevalence, individuals without symptoms of HCV infection often encounter false-positive anti-HCV test results in screening. The false positivity may be associated with other viral diseases genetically identical to the original infection-causing HCV strain, underlying autoimmune hepatitis, and a history of resolved or treated HCV disease. To reduce false-positive anti-HCV test results in populations with low HCV prevalence, the CDC has expanded the HCV diagnostic algorithm by providing anti-HCV S/Co values reflecting true antibody positivity for several manufacturers, allowing laboratories to create their own HCV diagnostic algorithms^2^. In this study, the relationship between anti-HCV and HCV-RNA positivity was retrospectively examined, aiming to determine the most suitable S/Co value for identifying true patients in anti-HCV testing.

In studies conducted in our country and worldwide, the most accurate S/CO value in anti-HCV ROC curve analysis has been found to be between 7.13 and 12.27^11-14^. In these studies, it has been suggested that a positivity below the S/CO value determined by ROC analysis may be a false positive, and it is recommended to repeat the anti-HCV test with a new sample at least 2 weeks later. In case of reactivity, it is further recommended to perform an HCV-RNA test.

In a retrospective study conducted in the United States, three different anti-HCV S/CO values were determined: 3, 8, and 20. The study recommended considering samples with an anti-HCV S/CO value <3.0 as true negatives, performing RIBA for those with an anti-HCV S/CO value between 3.0 and 19.9, conducting HCV RNA testing for RIBA-positive samples, and directly performing HCV RNA testing for samples with an anti-HCV S/CO value >20.0^15^.

In our study, the anti-HCV S/Co value was determined to be ≥10.86 in ROC curve analysis. No case with an S/CO value <3 was a true positive. Among cases with S/CO values between 3 and 7, two true patients had decompensated liver cirrhosis, and one of these patients had co-infection with HBV and HCV. It was considered that the low-titer level of anti-HCV could be secondary to HBV co-infection and/or decompensated liver cirrhosis. For patients with anti-HCV S/CO values between 7 and 10.86, false positives could be due to other viral diseases genetically identical to the HCV strain, and accordingly, we recommend repeating the anti-HCV test with a new sample at least 2 weeks later and, in case of reactivity, performing an HCV-RNA test. For patients with anti-HCV S/CO ≥10.86, it is considered appropriate to directly conduct an HCV-RNA test.

Furthermore, in our study, our recommendation is reinforced by the significantly higher anti-HCV levels in patients with positive HCV-RNA results compared to those with negative HCV-RNA results (p<0.0001) (Table 1), and by the significant correlation found in the Spearman correlation test between the anti-HCV S/Co ratio and HCV-RNA levels (p=0.013). We believe that an anti-HCV S/Co value ≥10.86 would be beneficial in detecting true patients.

In our study, the average age of patients was higher compared to other studies. Additionally, the average age of patients in the HCV-RNA positive group was statistically significantly higher than that of the HCV-RNA negative group (p=0.023)^4,12,13^. We believe that the presence of numerous elderly care centers in the location of our hospital and the conduct of pre-admission health screenings for these centers at our hospital may be the reason for this. Additionally, in other studies, foreign national patients were reported to be younger, and we consider this as another possible reason for the observed difference.

The hepatitis C virus has 8 main genotypes and 86 subtypes^16^. Genotype-1 is the most widespread globally. It is estimated that over one-third of genotype 1 cases are in East Asia, while three-quarters of genotype 3 cases are in South Asia. Genotype-4 is known to be prevalent in North Africa and Central Asia^17^.

In HCV genotyping studies conducted in our country, GT1 has been identified most frequently, with prevalence rates ranging from 65.1 to 88.4%^7,9,18^. Before 2010, GT1 rates were at their highest, but studies conducted after 2010 indicate a decline in GT1 rates. In studies conducted in our country over the past decade, an increase in GT3/4 and mixed genotypes has been emphasized, attributed to the higher inclusion of foreign national patients. Additionally, it has been highlighted that GT4 was first detected in Turkey in 2011, being the dominant genotype in Syria, and with the influx of refugees to Turkey, GT4 rates may have increased in recent years^7^.

The genotype distribution rates of the patients in our study are similar to the results of studies conducted in Turkey before 2010. Our GT3 rates were considerably lower compared to data from our neighboring country, Greece. GT4 was identified in four patients (3.07%), which is quite low compared to recent genotype study data in Turkey. Two patients of foreign nationality had a mixed genotype. The variation in genotype distribution in our study compared to other studies may be attributed to the location of our hospital on the Asian continent in Istanbul. The majority of residents in the vicinity of our hospital are typically local, while foreign national refugees tend to reside more on the European side of Istanbul. Recent HCV genotype studies in Turkey have mostly included data from hospitals on the European side of Istanbul, and these studies have particularly emphasized the inclusion of a high number of foreign national patients. In our study, patients with GT1 were found to be older, which was statistically significant. However, the younger age of foreign national patients and the predominance of GT3/GT4 patients could explain this age difference.

The retrospective nature of our study and the inability to evaluate transmission routes and risk groups are important limitations. Additionally, despite the large sample size, since the data represent the experience of a single center, the genotype distribution may not fully reflect the entire country. Some studies indicate that the sensitivity of the anti-HCV test is lower in patients undergoing hemodialysis due to low viral loads. Considering the possibility of patients with occult HCV infection among those undergoing hemodialysis and testing negative for anti-HCV, the prevalence of HCV found in our study might actually be higher.

## CONCLUSION

The study revealed a prevalence of 0.62% for HCV antibody positivity and a viremia prevalence of 0.13%. This rate was lower than the worldwide HCV viremia rate. In this study, if anti-HCV S/Co≥10.86 is detected, direct HCV RNA testing is recommended. For patients with S/Co values below 10.86, it should be considered that false positives may occur due to other viral diseases genetically identical to the HCV strain. In cases where high clinical suspicion for HCV persists, it is suggested to repeat the anti-HCV test with a new sample at least 2 weeks later, and if reactivity is confirmed for the second time, HCV RNA testing is recommended.
